# CO and CO_2_ daily time series and time series of wind speed

**DOI:** 10.1016/j.dib.2019.103976

**Published:** 2019-05-06

**Authors:** Changgong Shan, Wei Wang

**Affiliations:** a School of Environment Science and Optoelectronic Technology, University of Science and Technology of China, Hefei, 230000, China; b Key Laboratory of Environmental Optics and Technology, Anhui Institute of Optics and Fine Mechanics, Chinese Academy of Sciences, Hefei, 230031, China

## Abstract

The global CO_2_ has increased rapidly because of the vast use of fossil fuels over past 40 years. CO, co-emitted with CO_2_, also increased during this period. To understand the CO_2_ and CO regional emission, it is necessary to monitor the atmospheric CO_2_ and CO. Ground-based high-resolution Fourier transform infrared spectroscopy (FTIR), an important technique to observe atmospheric trace gases, is used to measure the column-averaged dry-air mole fractions (DMF) of CO2 and CO [1]. The DMF of CO and CO_2_ are not only insensitive to vertical diffusion, but also insensitive to the variation of surface CO_2_ and CO concentrations. Therefore, high-resolution Fourier transform spectrometer (FTS) is used to measure atmospheric CO_2_ and CO and obtain daily variation of CO_2_ and CO in Hefei site. A weather station was installed near the FTS to record the weather data. And the wind speed is related to turbulence. So the wind speed time series and Cumulative Distribution Function (CDF) are also shown in data article.

**Table:** Specifications Table

Subject area	*Physics, Environment science, Climate Science, Remote sensing*
More specific subject area	*Atmospheric remote sensing, Climate Science*
Type of data	*figure*
How data was acquired	*Record.*
Data format	*Cleaned raw data*
Experimental factors	*The ground-based high-resolution FTS is used to record the near-infrared solar spectra for retrieval of XCO*_*2*_*and XCO, and the wind speeds are recorded with the weather station.*
Experimental features	*The XCO*_*2*_*and XCO are integral values from the surface to the top of the atmospheric, and wind speed are recorded by an* anemometer.
Data source location	*Hefei, China. (31.9°, 117.71°)*
Data accessibility	*The data is with this article.*
Related research article	*Wang, W., Tian, Y., Liu, C., Sun, Y. W., Liu, W. Q., Xie, P. H., Liu, J. G., Xu, J., Morino, I., Velazco, V. A., G*rifﬁth, *D. W. T., Notholt, J., and Warneke, T.: Investigating the performance of a greenhouse gas observatory in Hefei, China. Atmos. Meas. Tech., 10, 2627–2643, 2017*[1]

**Table:** 

**Value of the data** • The data provide the time series and daily variation of the column-averaged dry-air mole fractions of CO2 and CO at Hefei site. • The researchers who study on the greenhouse gases, the greenhouse effect, the atmospheric constituents and how to validate the carbon satellite based on ground-based observations can benefit from this data. • The data can be downloaded from the TCCON data archive, or directly contact the authors to get them. The data can be used for further insights and development of experiments. • The time series and the cumulative distribution function of wind speed can be used to investigate the turbulence affects.

## 1 Data

**Fig. 1:**
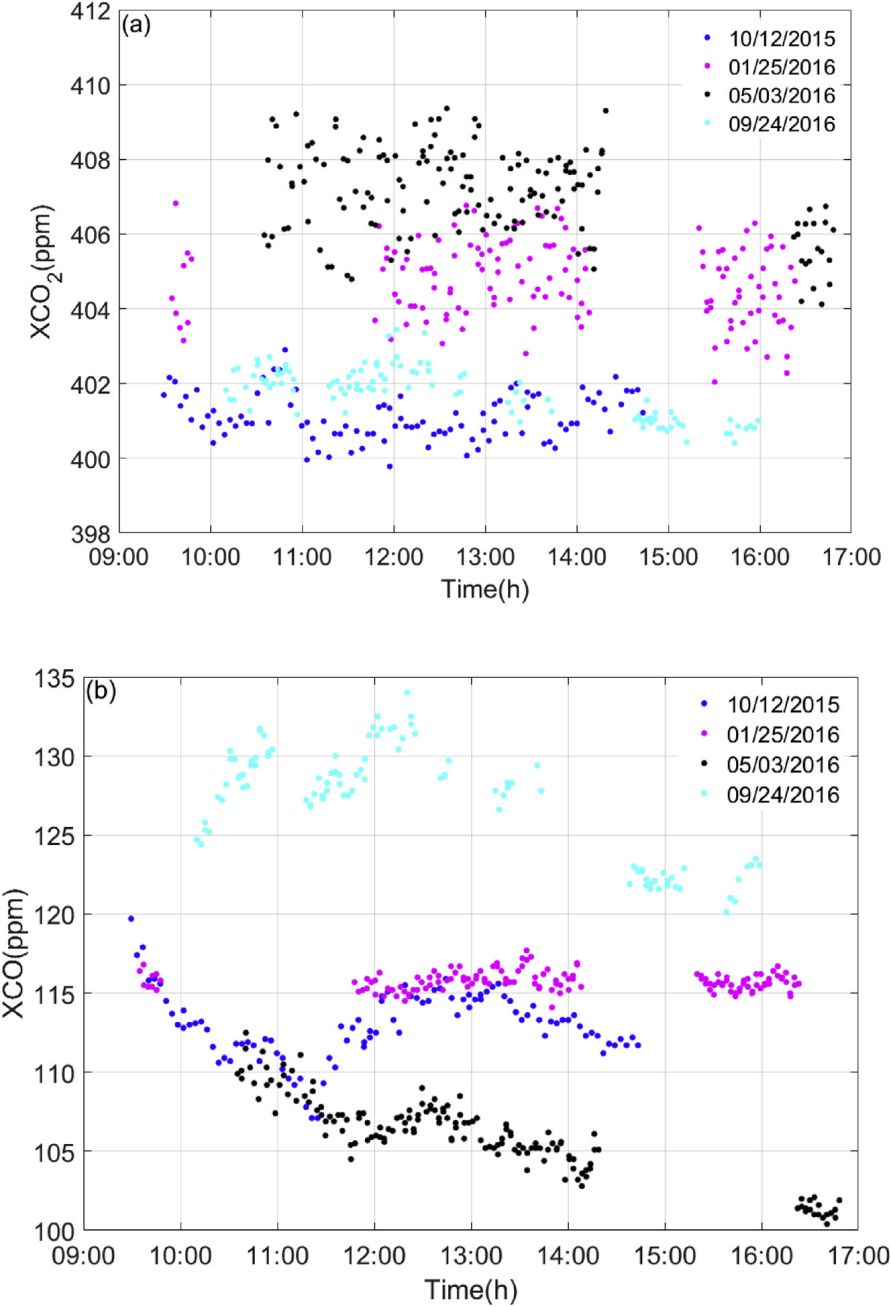
Daily variation of CO2 and CO in Hefei site. Time is local time in Hefei site.

**Fig. 2:**
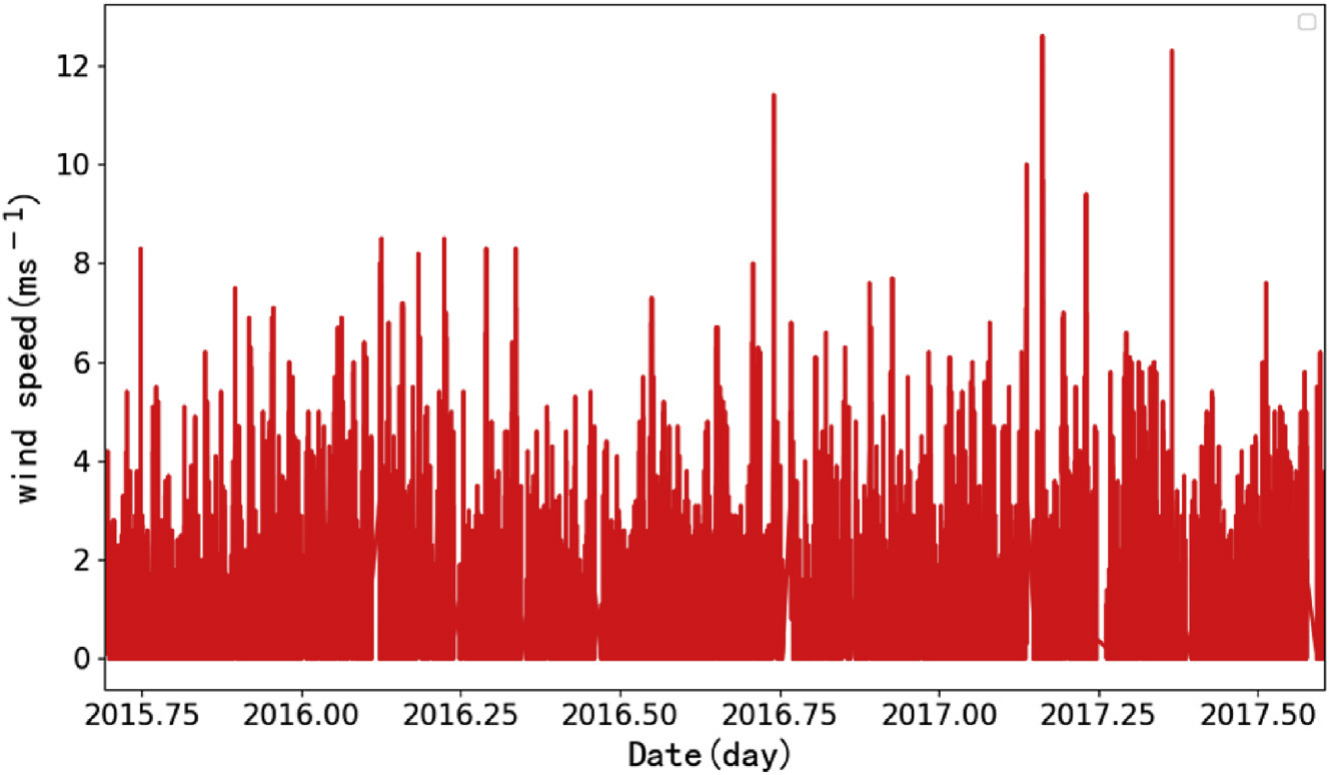
The time series of wind speeds in Hefei site.

**Fig. 3:**
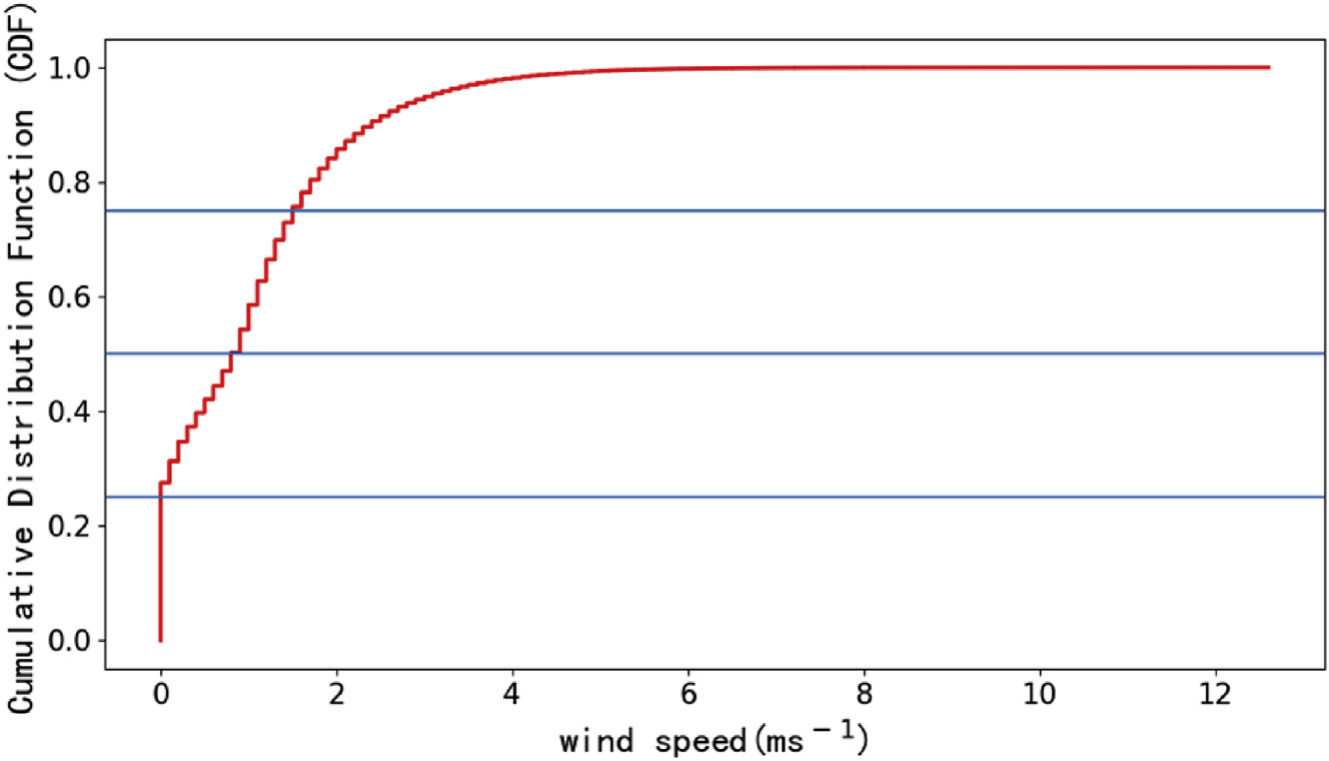
The statistics of Cumulative Distribution Function for wind speeds in Hefei site.

XCO_2_ and XCO data presented in the paper are observed from ground-based high-resolution FTS, and wind speed are recorded from weather station. The XCO_2_ and XCO are observed on four sunny days (Oct. 12, 2015, Jan. 25, 2016, May. 03, 2016 and Sep. 24, 2016) and the wind speeds are collected from Sep. 2015 to Aug. 2017. The XCO_2_ and XCO data in Fig. 1 are used to show how small diurnal variability of XCO_2_ after selection of spectra and this selection minimizes the air-mass-dependent biases. To investigate the effect of turbulence on measurement, the time series of wind speeds and the cumulative distribution function of wind speed during the observation period are also included in data. The data are shown in Fig. 2 and Fig. 3. Most wind speeds are lower than 4 m/s. Turbulence is insensitive to atmospheric stable condition [2], [3], [4]. There are two building around the observation site, one is 10 m high, and the other is 3 m high. The surrounding plants are sparse woods. And the turbulence intensity is weak and stable at the height of 4 m from canopy [5]. Therefore, the turbulence affects the measurements weakly. The raw data of this manuscript can be found in TCCON data from Hefei, China, Release GGG2014. R0, TCCON data archive, hosted by Caltech DATA, https://doi.org/10.14291/tccon.ggg2014.hefei01.R0,2018.

## 2 Experimental design, materials and methods

### 2.1 Observation site

Hefei site (31.9N, 117.17E, about 30 m above the sea level) is a continental site, and the site is located in the eastern China between the Yangtze River and Huai River. The site has four distinct seasons, and the annual average precipitation and average temperature are 1000 mm and 15 °C, respectively. To better understand the variation of CO_2_, CO and meteorological data in eastern China, one of the most industrialized regions in China, the ground-based high-resolution FTS solar spectrum observation system and weather station were installed in Hefei site.

### 2.2 Instrument setting

The ground-based remote sensing observation system consists of three parts: high-resolution FTS (Bruker IFS 125 HR), solar tracker (A547) and weather station (ZENO, Coastal Environmental Systems, USA). The solar tracker is used to track the sun, and reflects the solar beam into FTS. The FTS is used to collect the near-infrared solar spectra. In order to separate the spectral lines clearly, the spectral resolution is set 0.02cm^−1^. The FTS beamsplitter and detector are CaF_2_ and InGaAs, during observation period, respectively. To ensure the stability of the measurement, the instrument is evacuated under 10 hPa [1]. The measurements are on sunny days and sun intensity has no large fluctuations during spectra collection.

The weather station is installed near the solar tracker, equipped with temperature, humidity, surface pressure, wind speed and wind direction sensors to record the meteorological data. The weather station record the data every 10 s.

### 2.3 Data record

The XCO_2_ and XCO were retrieved from the high-resolution near-infrared solar spectra by the software GGG2014 [6]. The first step of retrieval is to exclude the bad spectra. The screening criteria are based on the solar intensity and the variation of solar intensity during measurement period. The spectral windows of CO_2_ and CO are from the Total Carbon Column Observing Network (TCCON) (https://tccon-wiki.caltech.edu/).

During solar spectrum measurement, the weather station also records the meteorological data. The meteorological data are input parameters in calculation of the atmospheric XCO_2_ and XCO.

The XCO_2_ and XCO calculated from the solar spectra are shown in Fig. 1. And the wind speeds recorded from the weather station are shown in Fig. 2.
