# Supplementary material for: CO and CO2 daily time series and time series of wind speed
Source: Data Brief. 2019 May 6;24:103976. doi: 10.1016/j.dib.2019.103976 (PMC6525326; doi:10.1016/j.dib.2019.103976)
Supplement: Multimedia component 1 [file mmc1.docx]

 Conflict of Interest

There are no conflicts of interest
